# A Large Intra-Abdominal Hiatal Hernia as a Rare Cause of Dyspnea

**DOI:** 10.1155/2015/546395

**Published:** 2015-07-01

**Authors:** Cem Sahin, Fatih Akın, Nesat Cullu, Burak Özseker, İsmail Kirli, İbrahim Altun

**Affiliations:** ^1^Department of Internal Medicine, School of Medicine, Mugla Sıtkı Kocman University, Orhaniye Mahallesi İsmet Catak Caddesi, Merkez, 48000 Mugla, Turkey; ^2^Department of Cardiology, School of Medicine, Mugla Sıtkı Kocman University, Orhaniye Mahallesi İsmet Catak Caddesi, Merkez, 48000 Mugla, Turkey; ^3^Department of Radiology, School of Medicine, Mugla Sıtkı Kocman University, Orhaniye Mahallesi İsmet Catak Caddesi, Merkez, 48000 Mugla, Turkey; ^4^Department of Gastroenterology, School of Medicine, Mugla Sıtkı Kocman University, Orhaniye Mahallesi İsmet Catak Caddesi, Merkez, 48000 Mugla, Turkey

## Abstract

Giant hiatal hernias, generally seen at advanced ages, can rarely cause cardiac symptoms such as dyspnea and chest pain. Here, we aimed to present a case with a large hiatal hernia that largely protruded to intrathoracic cavity and caused dyspnea, particularly at postprandial period, by compressing the left atrium and right pulmonary vein. We considered presenting this case as large hiatal hernia is a rare, intra-abdominal cause of dyspnea.

## 1. Introduction

Hiatal hernia is defined as abnormal protrusion of stomach with another intra-abdominal organ, in some cases, above diaphragm from esophageal hiatus. Its prevalence has been reported as 0.8–2.9% in upper gastrointestinal endoscopy series. Symptoms are often* related to* gastroesophageal reflux disease in the hiatal hernia which is usually asymptomatic. However, although rarely seen, a hiatal hernia can cause atypical symptoms such as chest pain or dyspnea due to the extent of hernia and organs protruded into thorax cavity. Here, we aimed to present a case with a hiatal hernia that largely protruded into thorax cavity and compressed left atrium, causing dyspnea.

## 2. Case Presentation

**Figure 1:**
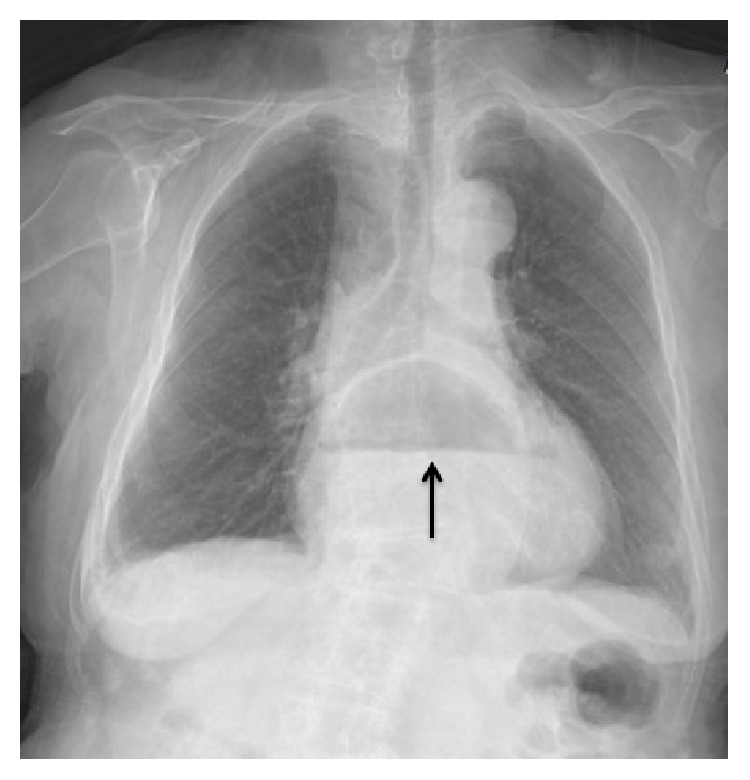
Appearance of air-fluid level superposed with cardiac contour on posterioanterior chest radiograph.

**Figure 2:**
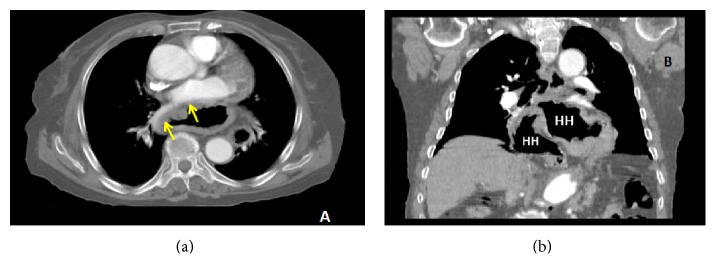
Axial and coronal images of the hiatal hernia on thoracoabdominal CT scan. (a) Compression to left atrium and right pulmonary vein by hiatal hernia on axial plane (yellow arrow); (b) herniation of majority of stomach into thorax cavity on coronal plane; HH: hiatal hernia.

*An 84-year-old woman* presented to the outpatient clinic with increasing fatigue, shortness of breath, and blackening of the stool over 1 month. She noted that shortness of breath aggravated with exertion and after the ingestion of food. The patient did not describe an underlying chronic disease and did not use any medication within the previous 6 months. On the physical examination, vital signs were stable and pallor was observed at conjunctiva. No abnormal physical examination finding was observed in the patient other than systolic murmur at apex on the cardiac examination. In the laboratory evaluations, the following results were obtained: WBC, 7400/mm^3^; Hgb, 10.9 g/dL; MCV, 72.3 fL; Plt, 376.000/mm^3^; iron, 18 *μ*g/dL; iron binding capacity, 350 *μ*g/dL; ferritin, 3,7 ng/mL. Fecal occult blood test was positive. Biochemical parameters were found to be within the normal range. On the posterioanterior chest radiograph, increased cardiothoracic index, enlarged mediastinum, and a mass appearance with an air-fluid level superposed with cardiac contours were observed (Figure 1). A thoracoabdominal CT scan including axial and coronal sections was performed in the patient because of the suspicion of a large hiatal hernia with available image. It was found that a large part of the stomach was herniated into mediastinum without any finding of incarceration and gastrointestinal obstruction and that hiatal hernia* compressed the* posterior wall of the left atrium and the right pulmonary vein (Figure 2).

**Figure 3:**
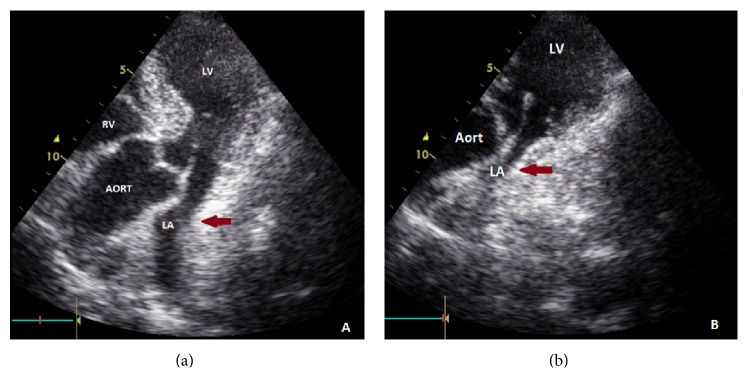
Transthoracic echocardiographic images of left atrial compression. Extrinsic compression to posterior wall of left atrium by hiatal hernia (red arrow). (a) Transthoracic echocardiographic view after fasting; (b) increased sol atrial compression on postprandial transthoracic echocardiography (red arrow); RV: right ventricle; LV: left ventricle; LA: left atrium.

On the upper gastrointestinal endoscopy, there were linear erosions at esophagogastric junction where hiatal hernia and diaphragmatic compression occurred. Active gastrointestinal bleeding was not observed.* Colonoscopy was considered normal*, which was performed for the etiology of iron deficiency anemia. On the transthoracic echocardiography, it was found that the left ventricle systolic and diastolic functions were normal, while the posterior wall of the left atrium was severely compressed by a large mass (Figure 3(a)). Systolic pulmonary artery pressure was estimated as high (42 mmHg) on echocardiography. As there was an increase in dyspnea after heavy meals according to anamnesis, postprandial transthoracic echocardiography was performed which revealed* an increase in the compression of the left atrium due to the hiatal hernia* (Figure 3(b)). Postprandial increase in dyspnea was attributed to the pulmonary congestion caused by the compression to the left atrium and the right pulmonary vein. Nutritional recommendations were given to the patient who denied surgical intervention due to the advanced age. A proton pump inhibitor and a motility regulator were prescribed. Hemogram was found to be normal 3 months after iron reemplacement therapy and the patient had an uneventful course.

## 3. Discussion

Hiatal hernia is defined as abnormal protrusion of stomach with another intra-abdominal organ, in some cases, above diaphragm from esophageal hiatus [1]. Type I hiatal hernia (sliding type) is the most commonly observed type in which gastroesophageal junction slides together with a part of the stomach [2]. Although the cause for the development of hiatal hernia is unknown, its incidence increases by advancing age [3]. It is accepted that relaxation at diaphragmatic crura resulting from aging process is the cause for the observation of more frequent and larger hiatal hernias in elder population [4].* Symptoms are often related to gastroesophageal reflux disease* in hiatal hernias which is usually asymptomatic. Generally, older people are unable to describe typical reflux symptoms such as burning at chest, acid regurgitation, and epigastric pain. Gastrointestinal bleeding related to ulcer or erosion, iron deficiency anemia, mucosal prolapse, incarceration, and volvulus are the main complications of hiatal hernia. Particularly, the most frightening complications are the development of incarceration or volvulus [2].

Iron deficiency anemia is one of the commonly seen complications in the setting of hiatal hernia.* Association between the* iron deficiency anemia and hiatal hernia has been known since the early 1930s [5].* The main reason for* iron deficiency anemia in hiatal hernia is the hemorrhage resulting from linear ulcers and erosions (Cameron lesion) at mucosal folds where diaphragmatic compression occurs [6, 7].* Bleeding is not the only factor* responsible for the anaemia in patients with hiatal hernia.* One of the reasons for iron deficiency anemia* in hiatal hernia is chronic gastritis with all the consequences. Today, although it is often missed, these lesions, one of the occult reasons for both gastrointestinal bleeding and iron deficiency anemia [8], are reported in 5% of the patients with hiatal hernia and 20% of the patients with persistent anemia and recurrent bleeding [9]. Bernardo et al. stated that Cameron lesions were not an uncommon cause of chronic gastrointestinal bleeding and should be kept in mind in the study of patients with iron deficiency anemia [10]. In our case, it was thought that chronic iron deficiency anemia had been explained by the advanced age and impaired oral ingestion in the prior examinations, resulting from occult hemorrhage secondary to hiatal hernia.

Although large hiatal hernias are infrequent, they can lead to atypical symptoms such as chest pain and dyspnea and rare complications such as pulmonary edema and cardiac failure due to the extent of hernia and the compression to heart and pulmonary veins by organs protruded into thorax cavity. Siu et al. reported that a large hiatal hernia caused cardiac failure by the compression to the left atrium in a case presenting with recurrent acute cardiac failure attacks [11]. In another case report, Chau et al. demonstrated a large hiatal hernia as the cause of chest pain in a patient that presented to emergency department with acute angina [12]. Hiatal hernia can manifest as a left atrial mass on echocardiography. It can cause pulmonary edema and cardiac failure through the pulmonary venous obstruction [13].

In our case, chronic fatigue and exertion dyspnea particularly aggravated at postprandial period. Increased pulmonary artery pressure in our case was attributed to the compression to the left atrium and the right pulmonary vein, as there was no other apparent cause. By available findings, it was thought that dyspnea aggravating after heavy meals is* due to pulmonary congestion from* the compression to the left atrium and the right pulmonary vein.

## 4. Conclusion

In conclusion, large hiatal hernias should be considered in the differential diagnosis as a rare intra-abdominal cause of persistent iron deficiency anemia and dyspnea. It should be kept in mind that large hiatal hernias can lead to cardiac symptoms and complications due to compression. Cases with* large hiatal hernias should be assessed by physical examination and imaging modalities such as echocardiography*.

## References

[1] Johnson D. A., Ruffin W. K. (1996). Hiatal hernia. *Gastrointestinal Endoscopy Clinics of North America*.

[2] Maziak D. E., Todd T. R. J., Pearson F. G. (1998). Massive hiatus hernia: evaluation and surgical management. *Journal of Thoracic and Cardiovascular Surgery*.

[3] Yoshimura M., Nagahara A., Ohtaka K. (2008). Presence of vertebral fractures is highly associated with hiatal hernia and reflux esophagitis in Japanese elderly people. *Internal Medicine*.

[4] Cole T. J., Turner M. A. (1993). Manifestations of gastrointestinal disease on chest radiographs. *Radiographics*.

[5] Bock A. V., Dulin J. W., Brooke P. A. (1933). Diaphragmatic hernia and secondary anemia: 10 cases. *The New England Journal of Medicine*.

[6] Windsor C. W., Collis J. L. (1967). Anaemia and hiatus hernia: experience in 450 patients. *Thorax*.

[7] Cameron A. J. (1976). Incidence of iron deficiency anemia in patients with large diaphragmatic hernia: a controlled study. *Mayo Clinic Proceedings*.

[8] Kimer N., Schmidt P. N., Krag A. (2010). Cameron lesions: an often overlooked cause of iron deficiency anaemia in patients with large hiatal hernias. *BMJ Case Reports*.

[9] Cameron A. J., Higgins J. A. (1986). Linear gastric erosion. A lesion associated with large diaphragmatic hernia and chronic blood loss anemia. *Gastroenterology*.

[10] Bernardo R. J., Portocarrero J. P., Tagle M. (2012). Cameron lesions: clinical experience. *Revista de Gastroenterología del Perú*.

[11] Siu C.-W., Jim M.-H., Ho H.-H. (2005). Recurrent acute heart failure caused by sliding hiatus hernia. *Postgraduate Medical Journal*.

[12] Chau A. M. T., Ma R. W.-L., Gold D. M. (2011). Massive hiatus hernia presenting as acute chest pain. *Internal Medicine Journal*.

[13] Lim H. S., Leong D. P., Alasady M. (2013). Massive hiatus hernia mimicking a left atrial mass. *Heart, Lung and Circulation*.

